# Etiology of Drug-Induced Edema: A Review of Dihydropyridine, Thiazolidinedione, and Other Medications Causing Edema

**DOI:** 10.7759/cureus.53400

**Published:** 2024-02-01

**Authors:** Evan S Sinnathamby, Bretton T Urban, Robert A Clark, Logan T Roberts, Audrey J De Witt, Danielle M Wenger, Aya Mouhaffel, Olga Willett, Shahab Ahmadzadeh, Sahar Shekoohi, Alan D Kaye, Giustino Varrassi

**Affiliations:** 1 School of Medicine, Louisiana State University Health Sciences Center (LSUHSC) New Orleans, New Orleans, USA; 2 School of Medicine, Louisiana State University (LSU) Health, Shreveport, USA; 3 School of Medicine, The University of Arizona College of Medicine - Phoenix, Phoenix, USA; 4 Department of Anesthesiology, Louisiana State University (LSU) Health, Shreveport, USA; 5 Department of Pain Medicine, Paolo Procacci Foundation, Rome, ITA

**Keywords:** steroids, nitrates, nsaids, ace-inhibitors, dihydropyridine, thiazolidinedione, edema

## Abstract

Edema is an accumulation of fluid in the body's tissues that affects millions of Americans yearly. It can affect multiple body parts, for example, the brain or eyes, but often occurs in the periphery, including the feet and legs. Medications, such as dihydropyridine and thiazolidinediones (TZDs), can be the etiology of edema. Edema can develop in association with problems in the vasculature or lymphatic flow. In recent years, a better understanding of these drug-induced mechanisms has been appreciated. Specifically, dihydropyridines can increase hydrostatic pressure and cause selective pre-capillary vessel vasodilation. TZDs can cause edema through increased vascular permeability and increased hydrostatic pressure. Specifically, peroxisome proliferator-activated receptor gamma (PPARγ) stimulation increases vascular endothelial permeability, vascular endothelial growth factor (VEGF) secretion, renal sodium, and fluid retention. Other drugs that can cause edema include neuropathic pain agents, dopamine agonists, antipsychotics, nitrates, nonsteroidal anti-inflammatory (NSAIDS), steroids, angiotensin-converting enzyme (ACE) inhibitors, and insulin. There are various clinical presentations of edema. Since multiple mechanisms can induce edema, it is important to understand the basic mechanisms and pathophysiology of drug-induced edema. Edema can even become fatal. For example, angioedema can occur from ACE inhibitor therapy. In this regard, it is considered a medical emergency when there is laryngeal involvement. This review aims to thoroughly appreciate the multiple causes of drug-induced edema and the ways it can be treated or prevented.

## Introduction and background

Edema is defined as swelling caused by the accumulation of fluid trapped in tissues. Over 200 million people worldwide experience edema every year [1]. Specifically, there is an association between edema and older age, female gender, minority race, low wealth, obesity, diabetes, hypertension, pain, low activity, and mobility limitations [1]. It can affect any body part, such as the brain, eyes, and lungs, but most often occurs in the legs and feet. This is generally termed peripheral edema. Chronic peripheral edema can cause pain, weakness, heaviness, discomfort, decreased range of motion, and a negative body image [2]. People may develop edema for multiple reasons, including impaired lymphatic drainage, increased capillary permeability, decreased oncotic pressure/hypoparaproteinemia, and increased hydrostatic pressure. Heart failure, diabetes, certain medications, and pregnancy are known common causes of edema. Untreated edema can cause intense pain and an increased risk of infection and ulceration [3]. There are many different ways to treat edema. In the case of edema caused by some underlying disease state, the etiology of the disease is treated. C1 esterase inhibitor concentrate is the treatment for edema secondary to hereditary angioedema, as a lack or dysfunction of the C1-inhibitor protein causes the autosomal dominant disease. For peripheral edema, certain diuretics can be given to alleviate swelling, but this often comes at the cost of frequent urination, which can lead to dehydration and decreased renal function [1,4]. Diuretics alongside osmotherapy, corticosteroids, and surgical decompression can be used to treat cerebral edema [5]. In the case of macular edema, corticosteroids and immunomodulators can be used as treatment [6].

Certain medications can also cause edema. In recent years, there has been an improved understanding of the various mechanisms that lead to the development of edema. Edema can become life-threatening when the pharynx or larynx is involved. Drug-induced angioedema has been reported in the use of beta-lactam antibiotics, non-steroidal anti-inflammatory drugs (NSAIDs), and angiotensin-converting enzyme (ACE) inhibitors [7]. Angioedema caused by ACE inhibitors is usually never associated with urticaria. It can occur years after the initiation of therapy and may happen irregularly with the use of ACE inhibitors. Maintaining airway patency is the most important requirement for patients who present with drug-induced angioedema. Medications can be used prophylactically to prevent exacerbations from occurring. Commonly, drug-induced edema presents as peripheral edema. Drug-induced bilateral pitting peripheral edema represents a polymorphic iatrogenic entity due to its mechanisms, severity, and clinical presentation [8]. It can also present as erythematous, unilateral, or petechial edema. There are also several mediators of drug-induced edema, like bradykinin, histamine, or leukotrienes. While this occurs frequently, drug-induced edema is still poorly characterized and underdiagnosed, which can lead to a prescribing cascade [8].

Therefore, this review aims to characterize the etiology of edema caused by certain medications. Emphasis will be placed on edema induced by dihydropyridine (DHP) and thiazolidine (TZD). Another section of our investigation includes other causes of drug-induced edema, which describes neuropathic pain agents, dopamine agonists, antipsychotics, nitrates, NSAIDS, steroids, ACE inhibitors, and insulin. NSAIDs specifically cause vasoconstriction by preventing the release of prostaglandins which act as vasodilators to the afferent arterioles in the kidneys. This leaves vasoconstrictors like angiotensin II, and catecholamines unopposed. We hope clinicians can use the present investigation to identify common symptoms of drug-induced edema, how severe the presentation is, and ways to treat edema based on underlying mechanisms.

## Review

General etiology of edema

There are four main causes of edema: increased capillary pressure, decreased oncotic pressure, increased capillary permeability, and impaired lymphatic drainage. Many disease states, such as cardiac failure, renal dysfunction, deep vein thrombosis, and cirrhosis, can increase capillary pressure. Capillary pressure is regulated on the arterial side by precapillary sphincters that can determine how much arterial pressure is put on the capillaries [9]. The venous side of the capillaries has poor regulation. As a result, when there are venous changes in pressure (i.e., blood volume expansion or venous blockage), there are also changes in capillary hydraulic pressure that can lead to edema [10-13].

Hypoalbuminemia is a major cause of reduced colloid oncotic pressure. Colloid oncotic pressure can be considered a pressure that keeps fluid in capillaries. It opposes interstitial oncotic pressure, which can filter fluid into the interstitial space. If colloid oncotic pressure is reduced, a net force pulls fluid out of the capillaries, which can cause fluid accumulation in the interstitial space. This can occur in several disease states, such as nephrotic syndrome, diabetic nephropathy, lupus nephropathy, amyloidosis, HIV-associated nephropathy, IgA nephropathy, cirrhosis, chronic liver disease, and malabsorption/malnutrition [9].

Increased capillary permeability is typically related to a vascular injury [9]. Injured vessels have increased porosity of their walls, which can lead to a net filtration of fluid out of the capillaries. The coefficient of proteins across the capillary wall also decreases, which can narrow the difference between the oncotic pressure of the capillary and the oncotic pressure below the endothelial glycocalyx. In effect, there is a reduced oncotic pressure gradient favoring fluid filtration. Capillary permeability can also increase in the setting of burn patients. This is related to the release of histamine and oxygen-free radicals, which can injure the endothelial lining of the microvasculature [14]. Some disease states, such as diabetes mellitus, can also cause injury to small and large blood vessel walls [9].

Lymphatic obstruction can also lead to edema. This is commonly seen in patients with lymphedema, tumors, fibrosis inflammation, certain infections, surgery, and congenital anomalies. Certain conditions, such as thyroid abnormalities, can cause an increase in interstitial albumin and other proteins, which can increase fluid filtration without an increase in lymphatic flow [9]. It is suggested that this is related to filtered proteins binding to interstitial mucopolysaccharides and preventing removal by the lymphatics [15]. Overall, there are several reasons edema can occur. The reason it occurs depends on the specific underlying pathophysiology.

All of these aforementioned changes can cause drug-induced edema. Clinicians must be informed about how certain medications can alter human physiology and cause edema. For example, DHP calcium channel blockers (CCBs) are a common way to treat hypertension (HTN), and TZD medications are one strategy to treat diabetes mellitus. However, the major adverse effect of both medications is peripheral edema. The pathophysiology of how these drugs can cause edema and how it can be treated will be discussed in the following sections. This investigation also describes other causes of drug-induced edema and how they can be treated as well.

DHP treatment and edema

Physiology of DHP in Treating HTN

CCBs stand as a vital class of drugs employed in the management of HTN [16]. Their distinct advantages include once-daily dosing and a reduced risk of metabolic abnormalities [17]. Functioning by binding to L-type voltage-gated calcium channels in both the vascular smooth muscle and the heart, CCBs effectively inhibit calcium influx into the cells. There are two primary categories of CCBs: DHPs and non-DHPs. While both categories act on vascular smooth muscle, non-DHPs also target the sinoatrial and atrioventricular nodes of the heart [18].

However, it is crucial to note that the use of CCBs, especially DHPs, is not without its challenges. A common adverse side effect associated with these medications is peripheral edema, which often results in the discontinuation of treatment by a significant number of patients [17].

Etiology of DHP-Induced Edema

Peripheral edema emerges as the most prevalent dose-dependent adverse effect of CCBs [19]. This effect is primarily attributed to the vasodilatory mechanism of CCBs on the vasculature. They tend to preferentially dilate pre-capillary vessels over post-capillary vessels, consequently elevating intracapillary pressure and leading to fluid extravasation into the interstitial space [20-29]. Additionally, CCBs may further exacerbate this effect by antagonizing the postural vasoconstrictor reflex [23,30]. It is important to highlight that this adverse effect is not a result of salt and water retention, as CCBs exhibit natriuretic properties [31,32]. The exact role of CCBs in altering capillary permeability as a contributor to edema remains unknown [33].

Clinical Manifestations and Targets

A meta-analysis conducted in 2011 revealed that approximately 25% of patients using CCBs experienced peripheral edema, leading to a withdrawal rate of 25% due to this side effect. Notably, CCB use was associated with an almost 11 times greater likelihood of peripheral edema [17].

Several factors influence the manifestation of edema induced by CCBs, including the severity variation among different CCBs [34-37]. DHP receptor CCBs, being more potent arteriolar dilators than non-DHP CCBs, are more commonly associated with peripheral edema [38,39]. Newer lipophilic DHP CCBs exhibit lower rates of peripheral edema compared to their predecessors [17].

Edema severity is dose-dependent and tends to increase with the duration of therapy. Additionally, environmental factors such as standing upright and exposure to warmer temperatures may modify edema manifestation. Other potential risk factors for CCB-induced edema include female gender, obesity, and older age [26,40-44].

Management Strategies

Identifying modifying factors provides an opportunity for tailored interventions to address CCB-induced edema. While adding a diuretic has limited effectiveness on vasodilatory edema, adjusting the CCB dose, especially lowering it, may be a viable option. Peripheral edema rates are nearly three times higher in high-dose CCBs than in low-dose counterparts [17].

Targeting the renin-angiotensin-aldosterone system (RAS) has shown promise in treating CCB-related edema. Blocking the RAS system through ACE inhibitors or angiotensin-one receptor blockers (ARBs) induces post-capillary dilation, normalizing intracapillary pressure and reducing fluid extravasation into the interstitial space [20]. Combining CCBs with an ACE inhibitor or ARB may be a preferable approach to high-dose CCB monotherapy [21,45-49].

Furthermore, considering the variation in edema severity among different CCBs, transitioning to a lipophilic CCB has been shown to significantly reduce edema rates, possibly due to a more gradual onset [17,48,50,51]. Combination therapy of an ACE inhibitor or ARB with a lipophilic DHP CCB may further mitigate the risk of edema [51]. Additionally, second or higher-generation DHP CCBs, when used in combination with an ACE inhibitor or ARB, may reduce the likelihood of peripheral edema [40].

TZD treatment and edema

TZDs constitute a class of oral medications designed to address type II diabetes mellitus (T2DM) by enhancing the body's response to insulin, thereby lowering blood glucose levels [52]. Pioglitazone and rosiglitazone are the two specific drugs within this class available in the United States market [53]. T2DM, marked by the progressive loss of insulin secretion by pancreatic β-cells within the context of peripheral insulin resistance, leads to glucose intolerance and hyperglycemia [54-56]. TZDs operate by increasing peripheral tissue sensitivity to insulin, primarily through the activation of the nuclear receptor peroxisome proliferator-activated receptor gamma (PPARγ) [52,57].

Mechanisms of Action and Pleiotropic Effects

The proposed mechanism involves PPARγ agonists stimulating genes that regulate insulin activity, adipose cell differentiation, and aspects of lipid metabolism [58-60]. TZDs have demonstrated the ability to redistribute fatty acids and intracellular lipids into adipocytes, suppress peripheral adipocyte lipolysis, and enhance adipocyte differentiation. Beyond glucose control, TZDs exhibit multifaceted benefits, including mitigating dyslipidemia, inflammation, vascular remodeling, and ectopic fat accumulation [61,62,63].

Adverse Effects and the Issue of Fluid Retention

Despite their therapeutic efficacy, TZDs have experienced a decline in usage due to their association with adverse effects, particularly fluid retention and congestive heart failure [64,65,66]. A consensus statement by the American Heart Association revealed a nuanced incidence of peripheral edema: 3% to 5% with TZD monotherapy, 7.5% when combined with sulfonylureas, and 13.1% to 16.2% when combined with insulin [67].

Mechanisms of TZD-Induced Edema

Understanding the pathogenesis of TZD-induced edema is critical, with PPARγ being a central mediator. Proposed mechanisms include PPARγ-mediated increases in vascular endothelial permeability, elevated vascular endothelial growth factor (VEGF) secretion, and enhanced sodium and fluid retention in renal proximal tubules and collecting ducts [14,17]. This multifactorial interplay contributes to peripheral edema of the extremities, pulmonary edema, and diabetic macular edema [68-70]. The latter, linked to diabetic retinopathy and a leading cause of blindness in T2DM patients, underscores the significance of addressing TZD-induced edema [71].

Emerging Therapeutics: The Case of Lobeglitazone

The introduction of lobeglitazone in Korea represents a newer and more potent iteration of TZDs. While early clinical trials suggest fewer off-target side effects, such as bladder cancer or heart failure, due to increased binding affinity and specificity for PPARγ, the risk of edema remains comparable to other TZDs [72,73]. As TZD-induced edema is primarily mediated by the on-target effects of PPARγ, this development may not substantially mitigate edema risk [67,73].

Clinical Considerations and Limitations of TZD Use

Given the heightened risk of adverse effects, including edema, associated with TZD use, their application in clinical practice has been considerably restricted. The incidence of edema correlates with the underlying disease magnitude [65], emphasizing the need for cautious patient selection by clinicians aiming to leverage the myriad benefits of TZD therapy. Comprehensive risk-benefit assessments are crucial, considering both glycemic control improvements and potential adverse outcomes.

Other causes of drug-induced edema

When considering drug-induced edema, it is easiest to categorize whether the effect is related to increased hydrostatic pressure, increased vascular permeability, or decreased capillary oncotic pressure. Specifically, those that influence hydrostatic pressure can be further subdivided based on their vasodilation and blood volume effects. Vasodilation subsequently may stimulate the RAS to cause sodium and water retention and enhanced blood volume.

Neuropathic Pain Agents

Reducing neuronal transmission via calcium channel antagonism is a common target in managing pain. Commonly used agents targeting this pathway include gabapentinoids, such as gabapentin and pregabalin. Blockade of the α2δ-1 subunit of calcium channels by these agents produces edema in similar pathogenesis as non-DHP CCBs like amlodipine [74]. Related to these receptors on vascular smooth muscle and cardiomyocytes, this effect can present as isolated peripheral edema or acute heart failure in those with underlying cardiac pathology [75]. The usual onset for both adverse reactions is within the first month of initiation or dose escalation, and regression is commonly seen after de-escalation without the need for diuretics.

Dopamine Agonists

Regarding their agonistic effects on the D2 receptor, dopamine agonists are used as antiparkinsonian agents to reduce unwanted motor movements and delay levodopa initiation [76]. Additionally, nonspecific interactions with other receptors are attributed to various reported side-effect profiles. Drugs in this class can be further divided into ergot and non-ergot derivatives. Older ergot derivatives, such as bromocriptine, are closely associated with pulmonary edema in those with valvulopathies due to their exacerbation of regurgitation [77]. This effect is believed to be due to partial affinity for the 5-HT2B receptor. Stimulation of this receptor mediates fibrotic changes within these tissues. The proliferation of fibroblasts expands the extracellular matrix, thus leading to fibrosing and stiffening of the valve leaflet. Additionally, both ergot and non-ergot derivatives, such as pramipexole and ropinirole, have been reported instances of causing peripheral edema. This additional off-target effect is thought to be due to alpha-2 receptor agonism causing the reduced sympathetic tone to the vasculature. Those at higher risk for occurrence often have preexisting vasculature pathology, such as coronary or peripheral artery disease [78].

Antipsychotics

Grouped based on their similar effects on psychosis, drugs within the atypical antipsychotics class display a wide variety of receptor affinity profiles [79]. Those with the greatest affinity for the alpha-1 receptor are closely associated with peripheral edema related to a reduction in sympathetic tone driving forward blood flow. Due to common being encountered upon rapid or large dose escalations, it is recommended to titrate doses [80] gradually. If severe, switching to another drug within the class, rather than re-challenging with the same drug, is preferred due to the high recurrence rate.

Nitrates

Nitrates preferentially dilate the venous vasculature rather than the arteriole to cause vasodilation and reduce preload [81]. While this effect can be advantageous in pulmonary edema by reducing stress on the left ventricle, it can cause venous pooling as a side effect. By causing vasodilation, forward blood flow is reduced, and venous pooling occurs as blood is redistributed from central circulation to the more compliant venous circulation. 

NSAIDs

Prostaglandins are a diverse group of hormone-like lipid molecules produced by cyclooxygenase-1 (COX-1) and COX-2 enzymes. Acutely, they are inflammatory mediators made in response to infection. Chronically, they can have deleterious effects that result in pain and arthritis. In the vasculature, prostaglandins are vasoactive molecules that vasodilate to buffer excessive vasoconstriction caused by angiotensin II and catecholamines. This counterregulatory system can maintain blood flow to various organs, including the kidneys. Non-selective NSAIDs and selective COX-2 inhibitors, such as celecoxib, may be used to alleviate pain sensation, but they also have undesirable effects on kidney function and blood pressure. By preferentially vasoconstricting the afferent arteriole in the kidneys, these drugs reduce the glomerular filtration rate and stimulate renin-angiotensin-aldosterone system-mediated sodium and water retention. The net impact is increased blood volume and blood pressure, which may cause edema related to increased hydrostatic pressure. Although non-selective NSAIDs tend to have a greater impact on the development of HTN than non-selective COX-2 inhibitors, both were shown to be similar in the rates of edema development [82]. This duration and dose-dependent effect was reversed upon discontinuation of the drug and generally not seen unless there was a pre-existing renal or cardiac pathology. 

Steroids 

Steroidal drugs can be considered based on their relative corticosteroid, glucocorticoid, and mineralocorticoid activity. Corticosteroid activity is advantageous in inflammatory and edematous states, such as cerebral edema, due to its anti-inflammatory and immunosuppressive properties. Mineralocorticoid activity, conversely, can increase sodium and water retention by the kidneys, leading to enhanced blood volume. Due to the aldosterone escape phenomenon, those with adequate kidney and cardiac function can increase distal sodium delivery to equalize urinary sodium excretion and intake to avoid edema formation [83]. Fludrocortisone has the most mineralocorticoid activity, while dexamethasone and methylprednisolone have relatively negligible activity [84].

ACE Inhibitors

Angioedema (AE) is a rare complication that occurs in less than 1% of those treated with ACE inhibitor therapy [85]. Areas of well-demarcated, non-pitting edema of deep subcutaneous tissues characterize this manifestation. The most commonly involved tissues include the face, lips, tongue, and oropharynx [86]. The pathogenesis is believed to have reduced metabolism and accumulation of the vasoactive peptide Bradykinin. Increased concentrations of this peptide lead to increased vascular permeability and fluid extravasation. Though generally self-limiting, it is considered a medical emergency when airway obstruction occurs. Because it may occur at any time throughout therapy, ongoing monitoring should occur throughout treatment. If AE occurs, switching to an ARB has not been shown to have an increased risk of recurrence [85].

Insulin

Glucose is an osmotically active molecule within the vasculature that can create an oncotic gradient favoring intravascular fluid shifts. Furthermore, chronic hyperglycemia has also been shown to reduce vascular integrity, promoting extravasation. During initiation or intensification of Insulin therapy in those with chronic hyperglycemia, peripheral edema may ensue in a condition known as insulin edema syndrome [87]. This is usually a relatively benign condition that can be avoided by careful dosing considerations or treated conservatively with sodium restriction or diuretics. A more severe consequence of uncontrolled diabetes is diabetic ketoacidosis (DKA). Following aggressive insulin initiation in these patients, cerebral edema may occur, causing significant mortality in an estimated 25% of patients [88]. Treatment strategies that reduce the occurrence include early recognition through frequent neurological exams during insulin treatment and aggressive rehydration strategies (Table 1).

**Table 1:** Comparisons of the pharmacological mechanisms and etiology of edema of the common agents of drug-induced edema. DHP CCB, dihydropyridine calcium channel blocker; TZD, thiazolidinedione; VEGF, vascular endothelial growth factor; PPARγ, peroxisome proliferator-activated receptor gamma; NSAIDs, non-steroidal anti-inflammatory drugs; GFR, glomerular filtration rate; RAAS, renin-angiotensin-aldosterone system; ACE, angiotensin-converting-enzyme

Drug type	Mechanism of edema	Pharmacological mechanism
DHP CCBs	Increased hydrostatic pressure	Selective vasodilation of pre-capillary vessels causes increases in intracapillary pressures.
TZDs	Increased vascular permeability & increased hydrostatic pressure	PPARγ stimulation increases vascular endothelial permeability, VEGF secretion, and renal sodium and fluid retention.
Neuropathic pain agents	Increased hydrostatic pressure	Calcium channel blockade induces selective vasodilation of arterioles causing increases in intracapillary pressures.
Dopamine agonists	Increased hydrostatic pressure	Alpha-2 receptor mediates reduction of sympathetic tone causing arteriolar dilation.
Antipsychotics	Increased hydrostatic pressure	Antagonistic effects on alpha-1 receptors cause vasodilation.
Nitrates	Increased hydrostatic pressure	Preferential venous dilation leads to venous pooling.
NSAIDs	Increased hydrostatic pressure	Preferential inhibition of vasodilation of renal afferent arterioles leads to decreased GFR and stimulation of RAAS-mediated sodium and water retention.
Steroids	Increased hydrostatic pressure	Mineralocorticoid activity leads to renal salt and water retention.
ACE inhibitors	Increased vascular permeability	Reduced metabolism and accumulation of bradykinin causes increased vascular permeability and fluid extravasation.
Insulin	Decreased capillary oncotic pressure and increased vascular permeability	Rapid correction of hyperglycemia causes a loss of oncotic pressure; chronic hyperglycemia mediates vascular membrane damage and increased permeability.

Discussion

Related to nonspecific and wide-ranging levels of severity, edema can be a manifestation secondary to an underlying pathophysiology or iatrogenic. Whether it is life-threatening or indolent, all presentations have an unfavorable impact on quality of life at the minimum. On initial presentation, life-threatening causes must first be ruled out. A thorough cardiac and pulmonary exam should be conducted to rule out cardiac and pulmonary causes of peripheral edema. Following the physical exam indicated, labs should be utilized when indicated if other causes are suspected, such as nephrotic syndrome and liver disease. Once these emergent scenarios have been ruled out, the clinician should conduct a thorough medication to identify medication-induced edema. Generally, edema is most associated with initiation of drugs and will present acutely during this period. However, it is important to keep in mind the exceptions to this rule, such as ACE inhibitors. Understanding the mechanisms by which these drugs cause edema is as insightful as knowing how to treat their adverse effects. For example, drugs that cause edema secondary to reduced oncotic pressure, such as insulin and slow dose titrations, can help allow the vasculature to adapt efficiently to osmolarity changes. Whereas drugs that cause edema secondary to increased hydrostatic pressure, diuretics are more effective at acute management.

Regardless of the physiological reason for the edema, all management strategies should include either dose de-escalations according to tolerability or replacement of the offending agent in severe or refractory cases. If the drug is maintained, prescribing within recommended dosing ranges and less than recognized maximum doses aids in reducing the risk of this adverse effect. For instance, CCBs, one of the most widely accepted causes of drug-induced edema, have dosing parameters that are often limited by edema formation.

## Conclusions

This paper delves into two prominent classes of medications, DHP CCBs and TZDs, both acknowledged for their efficacy in treating hypertension and T2DM, respectively. However, their use is marked by the common adverse effect of peripheral edema. DHPs, acting as potent arteriolar dilators, induce vasodilation, which elevates intracapillary pressure and leads to fluid extravasation. The intricacies of DHP-induced edema, its clinical manifestations, and management strategies, including dose adjustment and combination therapy, are meticulously explored. On the other hand, TZDs, while effectively improving insulin sensitivity, are associated with fluid retention and peripheral edema. The paper outlines the mechanisms of TZD-induced edema, the emerging therapeutic option of lobeglitazone, and the clinical considerations and limitations of TZD use. Furthermore, the paper broadens its scope by examining other causes of drug-induced edema, encompassing various drug classes such as neuropathic pain agents, dopamine agonists, antipsychotics, nitrates, NSAIDs, steroids, ACE inhibitors, and insulin. Each drug class is scrutinized for its specific mechanisms contributing to edema, clinical manifestations, and potential management strategies.

In essence, this comprehensive exploration emphasizes the imperative role of healthcare professionals in understanding the intricate relationships between drug mechanisms and physiological responses. The nuanced insights provided herein equip clinicians with the knowledge necessary to navigate the challenges of drug-induced edema, enabling more informed decision-making in patient care and therapeutic interventions.
